# Transcriptome sequencing of *Eucalyptus camaldulensis* seedlings subjected to water stress reveals functional single nucleotide polymorphisms and genes under selection

**DOI:** 10.1186/1471-2164-13-364

**Published:** 2012-08-01

**Authors:** Bala R Thumma, Navin Sharma, Simon G Southerton

**Affiliations:** 1 CSIRO Plant Industry, Clunies Ross Street, Acton, ACT, 2601, Australia; 2 ITC India, R&D Centre, Peenya Industrial Area, No. 3, Bangalore, Karnataka, 560058, India

## Abstract

**Background:**

Water stress limits plant survival and production in many parts of the world. Identification of genes and alleles responding to water stress conditions is important in breeding plants better adapted to drought. Currently there are no studies examining the transcriptome wide gene and allelic expression patterns under water stress conditions. We used RNA sequencing (RNA-seq) to identify the candidate genes and alleles and to explore the evolutionary signatures of selection.

**Results:**

We studied the effect of water stress on gene expression in *Eucalyptus camaldulensis* seedlings derived from three natural populations. We used reference-guided transcriptome mapping to study gene expression. Several genes showed differential expression between control and stress conditions. Gene ontology (GO) enrichment tests revealed up-regulation of 140 stress-related gene categories and down-regulation of 35 metabolic and cell wall organisation gene categories. More than 190,000 single nucleotide polymorphisms (SNPs) were detected and 2737 of these showed differential allelic expression. Allelic expression of 52% of these variants was correlated with differential gene expression. Signatures of selection patterns were studied by estimating the proportion of nonsynonymous to synonymous substitution rates (Ka/Ks). The average Ka/Ks ratio among the 13,719 genes was 0.39 indicating that most of the genes are under purifying selection. Among the positively selected genes (Ka/Ks > 1.5) apoptosis and cell death categories were enriched. Of the 287 positively selected genes, ninety genes showed differential expression and 27 SNPs from 17 positively selected genes showed differential allelic expression between treatments.

**Conclusions:**

Correlation of allelic expression of several SNPs with total gene expression indicates that these variants may be the *cis*-acting variants or in linkage disequilibrium with such variants. Enrichment of apoptosis and cell death gene categories among the positively selected genes reveals the past selection pressures experienced by the populations used in this study.

## Background

Abiotic or environmental stresses such as drought, heat, salinity and cold are major impediments to plant survival and productivity in many parts of the world. Plants respond to abiotic stress conditions through diverse biochemical and physiological processes such as accumulation of osmolytes and proteins, reduction in stomatal conductance, increase in photorespiration and general reduction in growth rate. Osmotic adjustment is one of the common mechanisms of plant response to abiotic stress signals. Water availability is the limiting factor common to all abiotic stresses. As the water potential of the soil water decreases, plants accumulate solutes to reduce the osmotic potential and to maintain water uptake [1]. Several inorganic solutes such as K+, Na+, Cl- and organic solutes such as total soluble sugars, proline, glycine betaine and mannitol are involved in osmotic adjustment. Stress conditions also lead to accumulation of harmful reactive oxygen species (ROS) such as hydroxyl radicals, singlet oxygen, hydrogen peroxide (H_2_O_2_) and super oxide (O_2_-). Antioxidant enzymes such as superoxide dismutase (SOD), ascorbate peroxidase (ASX) and catalase help protect plants cells from the harmful effects of ROS [2]. Expression of several detoxification enzymes was shown to increase under stress conditions [3].

Several studies of transcriptional responses to abiotic stress using microarrays [4-8] have identified stress inducible genes that often belong to one of two groups, based on the functions of their gene products. Genes belonging to group 1 are mainly involved in water transport (aquaporins), cellular membrane protection and integrity under stress conditions (proline, glycine betaine, mannitol), scavenging of free oxygen radicals (SOD, catalase), and protecting macromolecules (late embryogenesis abundant proteins - LEA, chaperons). The second group consists of genes that encode regulatory proteins (transcription factors, protein kinases, protein phospatases and calmoduluin binding proteins) and proteins involved in signal transduction [9,10]. Stress induced transcription factors are classified into two classes, ABA dependent and ABA independent. The ABA dependent transcription factors include MYC/MYB and ABA responsive element binding/ABA binding factor (AREB/ABF) and the ABA independent transcription factors are dehydration responsive element binding/C-repeat binding factors (DREB/CBF) belonging to the ethylene responsive factor/APETALA2 (ERF/AP2) family [9,11]. The other transcription factors responding to abiotic stress conditions are basic-domain leucine-zipper (bZIP)[12], WRKY binding [13] and NACs [14].

While microarray analyses are useful in revealing genes that are responsive to different conditions, identification of allelic variants from genes showing differential expression may enable their application in breeding by marker-assisted selection. Recent developments in sequencing technology are making it possible to combine gene discovery with identification of allelic variation. Transcriptome sequencing or RNA sequencing (RNA-seq) is an approach for quantifying transcripts, in which RNA samples are converted to cDNA and sequenced, typically using high throughput methods. The resulting reads are then mapped against a reference genome sequence or assembled de novo to produce genome-scale transcriptome maps consisting of the structure and abundance of each gene [15]. The abundance of each transcript is determined by counting the number of sequences mapped to the corresponding gene thus providing digital estimate of gene expression. The main advantages of RNA-seq over microarray analysis are a). As RNA-seq is based on counting sequences, cross hybridisation problems associated with microarrays are avoided b). RNA-seq has high dynamic range of detection i.e. very low and very high abundance transcripts can be detected with RNA-seq while microarrays lack sensitivity to detect genes expressed at either high or low levels. Using this technique Zenoni *et al.*[16] detected several genes expressed during berry development in *Vitis vinifera*. Similarly several protein coding genes related to xylem formation were identified in an *Eucalyptus* plantation tree using RNA-seq [17]. RNA-seq is also useful for identifying and estimating transcript abundances from alternatively spliced variants [18]. By sequencing several individuals from different populations it is also possible to identify single nucleotide polymorphisms (SNPs) from genes showing differential expression.

In addition transcriptome sequencing can also be used to study the evolutionary selection patterns of genes by estimating nonsynonymous to synonymous substitution (Ka/Ks) ratios. Novaes *et al.*[19] have shown that most of the genes are under purifying selection by sequencing RNA from different tissues bulked from several individual trees in *E. grandis*. Combining gene discovery with analysis of selection signatures may provide insights into natural selection patterns under drought stress.

*Eucalyptus camaldulensis* is one of the most widely planted tree species in the world [20], and is grown extensively in plantations for pulp production in the tropics of South and South East Asia [21,22]. Water availability is the most important factor determining the establishment and composition of tree species in the dry tropics [23]. The seedling stage is the critical period for survival and establishment of trees [24]. In this study we analysed the physiological responses of seedlings of three *E. camaldulensis* populations subjected to water stress. RNA extracted from leaves of these seedlings was used in RNA-seq analysis to study gene expression patterns under well watered and water stressed conditions. The main objectives of this study are to identify genes differentially expressed under control and stress conditions, to identify allelic variants from these genes and to study the evolutionary signatures of selection.

## Results

### Effect of water stress on physiological traits

Effect of water stress on several physiological and growth traits was analysed by comparing well-watered and water-stressed plants. Two-way ANOVA revealed significant differences between control and stress treatments for all the physiological and biomass traits except for root to shoot ratios (Table 1). While the treatment effect was significant, the population effect was not significant for any of the traits. Similarly no significant interaction between the treatment and population was observed for any of the traits. Pair-wise comparisons between the populations for traits were also not significant (data not shown).

**Table 1:** Two-way analysis of variance

**Source of Variation**	**WP**	**OP**	**TP**	**SC**	**Transpiration**	**TDM**	**TE**	**R/S**
Population	0.4736	0.7144	0.4769	0.1398	0.9749	0.4925	0.4042	0.4597
Treatment	<0.0001	<0.0001	<0.0001	<0.0001	< 0.0001	<0.0001	0.0028	0.268
Interaction	0.6461	0.8215	0.3869	0.9773	0.1565	0.1855	0.1211	0.0983

*WP*-water potential; *OP*-osmotic potential; *TP*-turgor pressure; *SC*-stomatal conductance; *TDM*-total dry matter; *TE*-transpiration efficiency; *R/S*-root to shoot ratio. Sampling 2 (52 days after stress) water relations were analysed.

*P*-values of population, treatment - control vs stress and interaction effects from a two-way analysis of variance of traits.

### Water stress significantly affects Leaf water relations and stomatal conductance

**Figure 1:**
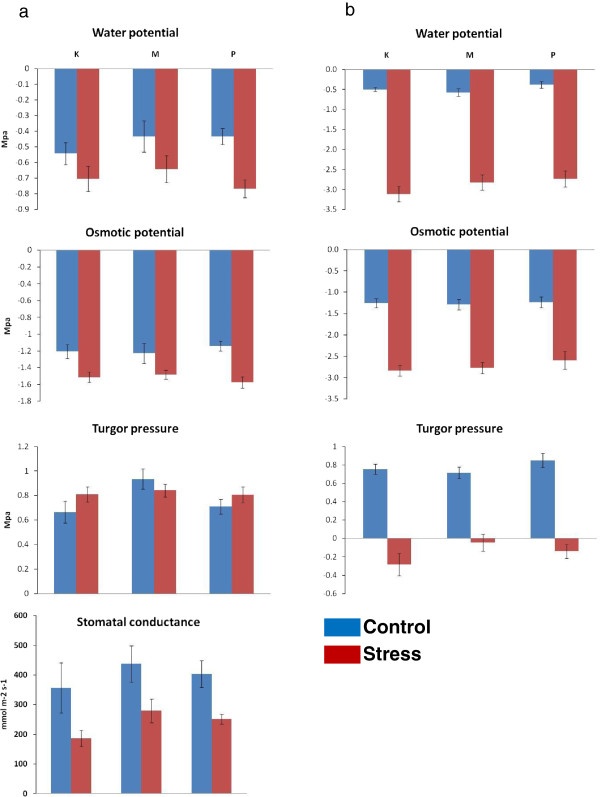
**Leaf water relations and stomatal conductance in response to water deficit. a)**. Readings taken 30 days after stress treatment (sampling 1); **b)**. Readings taken 52 days after stress treatment (sampling 2). Stomatal conductance was measured 10 days after stress treatment. Error bars are standard errors of mean (SEM).K–Katherine; M-Mt Isa; P-Petford.

Leaf water relations were measured on samples collected 30 days (sampling 1) and 52 days (sampling 2) after the imposition of stress treatment. Between the two sampling periods, measurements of water relations (pre-dawn water potential, osmotic potential and turgor pressure) were very similar in control seedlings (Figure 1a, b). However, in stressed seedlings highly significant differences were observed for these traits between the two sampling periods. Within a treatment at both sampling periods, no significant differences were observed between the populations for any of the water relation traits measured (Figure 1a, b). The differences between control and stressed seedlings were much more pronounced 52 days after the imposition of the stress treatment (sampling 2). After 30 days pre-dawn water potentials had decreased to −0.67 MPa in stressed seedlings compared to −0.47 MPa in controls. By 52 days pre-dawn water potentials had fallen to −2.89 MPa and negative turgor pressures were observed in stressed seedlings while in controls these traits were similar to those in sampling 1.

Mean stomatal conductance was higher in control seedlings (average: 399.45 mmol m^-2^ s^-1^) than in water stressed seedlings (average: 239.25 mmol m^-2^ s^-1^). Reduction in the stomatal conductance of the Katherine population is higher compared to the other two populations (Figure 1a), however, as with water relations, the stomatal conductance of the three populations were not significantly different (Table 1).

### Water stress significantly reduces biomass production under stress treatment

Water stress had a significant effect on all traits related to biomass production (Table 1). There was a significant decrease in the amount of water transpired and consequently there was a significant reduction in total dry mass (TDM) produced by stressed seedlings (Additional file 1). The amount of transpiration fell from 49.5 kg (control) to 14.0 kg under stress treatment and total biomass produced fell from 112.2 g (control) to 28.7 g under stress treatment. Similarly transpiration efficiency (TE-biomass produced per unit of water transpired) decreased from 2.24 g/kg in control seedlings to 2.00 g/kg in stressed seedlings. Root to shoot ratios of the Katherine and Mt. Isa populations increased while in the Petford population they decreased under stress treatment however, these differences were not significant (Table 1).

### RNA sequencing and differential gene expression

In total, 52 million reads were generated from 12 samples. Reads per sample ranged from two to nine million with an average of 4 million reads per sample. Reads from high throughput sequencing were analysed with TopHat package to develop gene models. Reference-guided mapping was used to predict gene models by mapping the reads against the *E. grandis* reference genome sequence without using *E. grandis* annotations. By using the coordinates from the predicted gene models we identified the *E. grandis* genes mapping to the predicted gene regions E. Camaldulensis'. While several of the predicted gene models map to *E. grandis* gene models there were however several predicted gene models E. Camaldulensis that did not map to *E. grandis* gene models. We used *E. grandis* gene names wherever the predicted models mapped to the *E. grandis* models. Where there are no *E. grandis* annotations mapping to the predicted gene models we used the gene names with a “CUFF” prefix. The coordinates of these genes are presented in Additional file 2.

### Reference-guided transcriptome mapping

**Figure 2:**
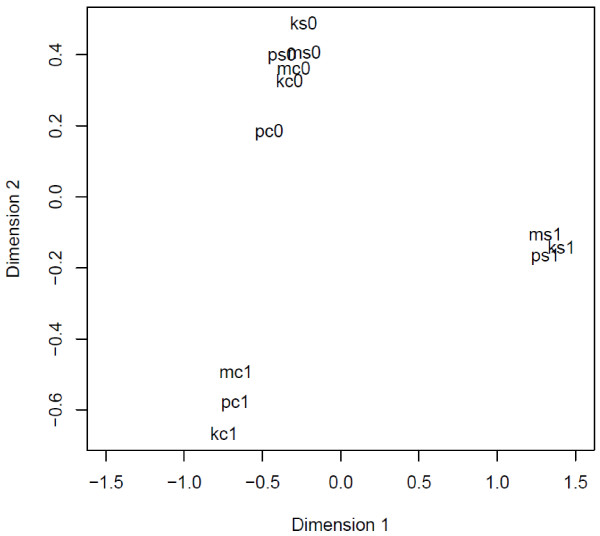
**Multi-dimensional scaling (MDS) plot of gene expression of 12 RNA-seq libraries.** S0 and C0 were samples collected at the beginning of the treatment. Control samples (C1) and stressed samples (S1) collected at the end of the treatment; K = Katherine, P = Petford, M = Mount Isa.

Reads from all the 12 libraries were mapped against the *Eucalyptus* reference genome sequence to generate gene annotations using the TopHat and Cufflinks packages. A total of 32,474 transcripts were predicted including a large number of alternatively spliced transcripts. The identity of the transcripts was investigated by BLAST searches against the *Arabidopsis* protein database. This analysis revealed 15,538 unique genes from the total transcripts. Read counts mapping to the gene annotations generated by reference-guided transcriptome mapping were used for testing differential expression of the genes between control (C1) and stress (S1) treatments using the edgeR package. Before testing for differential expression, diagnostic tests were performed to test the consistency of the data between the populations. A high correlation was observed in gene expression between the three populations from a given treatment as measured by the read counts. The Pearson’s correlation coefficient between the read counts of the three populations before stress treatment (total six libraries 3 from S0 and three from C0) ranged from 0.94 to 0.99 and the correlation coefficient between the three populations of control plants at the end of the experiment (C1) ranged from 0.93 to 0.95. Similarly in the stress treatment (S1) the correlation coefficients between the populations ranged from 0.94 to 0.97 (Additional file 3). This is further reflected in clustering analysis. Multi-dimensional scaling (MDS) plot of the count data clearly separated the 12 libraries into three groups (Figure 2). The six libraries from the three populations before treatment (S0 and C0) were clustered together. Similarly the three populations of control plants (C1) at the end of the treatment clustered together while populations from the stress treatment (S1) formed another cluster. As there is a high degree of similarity between the populations from a treatment, reads from each population from a treatment were used as biological replicates in testing for differential expression.

### Identification of genes responding to water stress conditions

**Figure 3:**
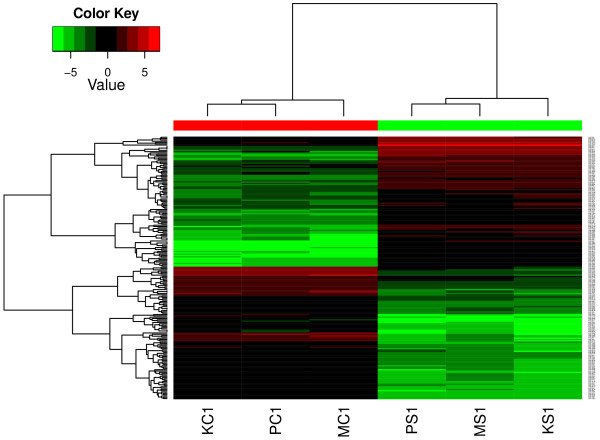
**Heatmap showing differential expression of top 200 genes between control (C1) and stress (S1) treatments.** Dark shades indicate higher expression and light shades indicate lower expression. Color key indicates the intensity associated with normalized expression values. Abbreviations as in Figure 2.

To identify genes responding to stress treatment, samples from control (C1) and stress (S1) treatments taken at the end of the stress treatment were analysed for differential gene expression. Analysis of differential gene expression revealed a total of 5270 transcripts (4320 unique genes based on *E. grandis* annotations) that were significantly (*P* ≤ 0.05; Bonferroni correction) differentially expressed between the control (C1) and stress (S1) treatments (Additional file 2). Read counts from the three libraries within each treatment are very similar (Table 2). A heatmap of gene expression of the top 200 genes is shown in Figure 3. Variance stabilized data obtained with DESeq pacckage was used to generate the heatmap. The gene expression patterns between the treatments are distinct while within each treatment they are similar. Gene identities of the most differentially expressed transcripts are shown in Table 3. Several heat shock proteins, cell wall genes such as expansins and drought stress related transcription factors (*HB-12*, *RD26*, *ERF110*) are among the most strongly differentially expressed genes.

**Table 2:** Read counts of significant genes under control and stress treatments

**Gene_id**	**KC1**	**MC1**	**PC1**	**KS1**	**MS1**	**PS1**	**logFC**	**FDR**
Eucgr.K02440	145	81	98	0	0	0	−35.5	2E-35
CUFF.8283	0	0	0	20	4	22	31.8	2E-06
Eucgr.L01022	0	1	1	82	3	161	5.8	8E-17
Eucgr.I02271	6	1	43	3025	1982	7510	7.1	1E-62
Eucgr.I01041	120	39	138	0	0	0	−35.3	3E-33
Eucgr.H04038	0	2	3	790	199	634	7.4	7E-44
Eucgr.J00639	2	2	6	248	187	563	5.8	5E-33
Eucgr.J00493	0	0	0	727	130	181	36.4	2E-43
Eucgr.H00163	4	0	1	382	218	461	6.9	6E-39
Eucgr.G01843	0	0	0	86	47	113	34.3	6E-22
Eucgr.A02965	10	44	91	0	0	0	−34.4	3E-23
Eucgr.F03575	89	17	40	0	0	0	−34.3	1E-22
Eucgr.F02915	26	25	90	0	0	0	−34.3	2E-22
Eucgr.F02733	0	1	0	60	96	38	7.1	3E-20
Eucgr.F02646	467	635	778	0	0	0	−38.1	2E-67
Eucgr.F01093	13	16	16	2534	973	2670	6.2	1E-49
CUFF.28412	0	0	0	843	400	1068	37.5	3E-56
Eucgr.F00644	2	11	10	4666	2578	10230	8.6	2E-75
Eucgr.F00195	41	44	17	0	0	0	−33.9	3E-19
Eucgr.E03257	1	1	3	251	142	436	6.5	3E-33

*M* = Mt.Isa; *K* = Katherine; *P* = Petford; *C1*- control; *S1*- stress; *logFC* – log fold change; *FDR*-false discovery rate.

Read counts of top 20 most differentially expressed transcripts under control (C1) and stress conditions (S1). *E. grandis* gene names are used when the predicted genes are mapped to *E. grandis* gene coordinates otherwise the predicted gene names are used with a pre-fix ‘CUFF’.

**Table 3:** Gene identities of the most differentially expressed transcripts

**Gene_id**	**LogFC**	**FDR**	**AT_accession**	**TAIR_gene_annotation**^**1**^
Eucgr.K02440	−35.5	2E-35	AT3G53190.1	Pectin lyase-like superfamily protein
CUFF.8283	31.8	2E-06		no-hit
Eucgr.L01022	5.8	8E-17	ATCG00020.1	photosystem II reaction center protein A
Eucgr.I02271	7.1	1E-62	AT3G12500.1	basic chitinase
Eucgr.I01041	−35.3	3E-33	AT3G10450.2	serine carboxypeptidase-like 7
Eucgr.H04038	7.4	7E-44	AT3G62420.1	basic region/leucine zipper motif 53
Eucgr.J00639	5.8	5E-33	AT5G21170.2	5'-AMP-activated protein kinase
Eucgr.J00493	36.4	2E-43	AT2G27510.1	ferredoxin 3
Eucgr.H00163	6.9	6E-39	AT2G38940.1	phosphate transporter 1;4
Eucgr.G01843	34.3	6E-22	AT5G45180.1	Flavin-binding monooxygenase
Eucgr.A02965	−34.4	3E-23	AT5G17050.1	UDP-glucosyl transferase
Eucgr.F03575	−34.3	1E-22	AT1G32860.1	Glycosyl hydrolase
Eucgr.F02915	−34.3	2E-22		no-hit
Eucgr.F02733	7.1	3E-20	AT1G78780.2	pathogenesis-related
Eucgr.F02646	−38.1	2E-67	AT5G09360.1	laccase 14
Eucgr.F01093	6.2	1E-49	AT4G27410.3	RD26 | NAC domain transcriptional regulator
CUFF.28412	37.5	3E-56	AT4G27450.1	Aluminium induced protein
Eucgr.F00644	8.6	2E-75		no-hit
Eucgr.F00195	−33.9	3E-19	AT5G42830.1	acyl-transferase
Eucgr.E03257	6.5	3E-33	AT2G33830.2	Dormancy/auxin associated
Eucgr.D02645	6.0	8E-47	AT3G61890.1	homeobox 12
Eucgr.D02610	−35.6	3E-36	AT1G73880.1	UDP-glucosyl transferase
Eucgr.D02390	−34.1	3E-21	AT5G49330.1	myb domain protein
Eucgr.A02214	−34.1	1E-20	AT1G28110.2	serine carboxypeptidase-like 45
Eucgr.A02209	−34.4	1E-23	AT1G28110.2	serine carboxypeptidase-like 45
Eucgr.A02209	−34.8	1E-27	AT2G33530.1	serine carboxypeptidase-like 46
Eucgr.D00606	7.2	1E-57	AT1G28330.4	dormancy-associated protein-like 1
Eucgr.A02070	5.8	5E-43	AT3G04070.1	NAC domain containing protein 47
Eucgr.C04029	−33.9	2E-19	AT2G24800.1	Peroxidase superfamily protein
Eucgr.C03801	−34.9	2E-28	AT1G67290.1	glyoxal oxidase-related protein
Eucgr.C03715	5.8	3E-17	AT5G56550.1	oxidative stress 3
Eucgr.C03715	6.7	5E-25		no-hit
Eucgr.A01963	−36.5	2E-47	AT2G42840.1	protodermal factor 1
Eucgr.C01020	−34.1	1E-20	AT5G58880.1	unknown protein
Eucgr.A01768	−35.3	6E-33	AT4G03270.1	Cyclin D6;1
Eucgr.B03987	6.4	5E-57	AT1G60470.1	galactinol synthase 4
Eucgr.B03602	−34.4	9E-24		no-hit
Eucgr.B01275	6.2	8E-20	AT5G18600.1	Thioredoxin superfamily protein
CUFF.12241	−35.7	3E-38		no-hit
Eucgr.L02741	−36.1	1E-42	AT5G17040.1	UDP-Glycosyltransferase superfamily protein
Eucgr.K01836	10.3	6E-88	AT5G06760.1	Late Embryogenesis Abundant 4-5
Eucgr.I02392	12.7	2E-85	AT2G37670.1	no-hit
Eucgr.C00146	−10.4	2E-84	AT2G22980.4	serine carboxypeptidase-like 13
Eucgr.F04160	−11.7	5E-83	AT5G09360.1	laccase 14
Eucgr.I01495	9.5	7E-79	AT3G12500.1	basic chitinase
Eucgr.I02395	7.8	7E-77	AT2G21490.1	dehydrin LEA
Eucgr.B02163	−9.5	3E-76	AT4G28780.1	GDSL-like Lipase/Acylhydrolase
Eucgr.H04427	−8.7	7E-74	AT3G06350.1	dehydroquinate dehydratase, putative / shikimate dehydrogenase, putative
Eucgr.E00358	9.2	2E-73	AT4G17030.1	expansin-like B1
CUFF.37010	8.6	4E-72	AT2G47770.1	TSPO(outer membrane tryptophan-rich sensory protein)
Eucgr.G01342	−7.6	3E-71	AT4G15480.1	UDP-Glycosyltransferase superfamily protein
Eucgr.L02980	7.8	2E-70	AT4G27670.1	heat shock protein 21
Eucgr.K00423	7.9	2E-70	AT4G27670.1	heat shock protein 21
Eucgr.C01021	−10.8	6E-68	AT5G17050.1	UDP-glucosyl transferase 78D2
Eucgr.I01044	−10.5	3E-67	AT2G22960.1	alpha/beta-Hydrolases
Eucgr.F03917	−11.7	3E-66		no-hit
Eucgr.I01037	−7.9	3E-66	AT1G33540.1	serine carboxypeptidase-like 18
Eucgr.G01113	−8.3	6E-66	AT1G73290.1	serine carboxypeptidase-like 5
CUFF.9597	11.1	2E-65	AT5G63350.1	unknown protein
Eucgr.H02828	−7.2	6E-65	AT5G13930.1	Chalcone and stilbene synthase
Eucgr.K02961	7.7	3E-64	AT2G40300.1	ferritin 4
Eucgr.C02812	−7.2	5E-64	AT5G11420.1	unknown
Eucgr.I01954	−11.7	2E-63	AT1G69530.5	expansin A1
CUFF.1110	−7.2	5E-63	AT4G16730.1	terpene synthase 02
Eucgr.A02982	8.0	1E-62		no-hit
Eucgr.A02983	7.5	1E-62		no-hit
Eucgr.B00944	−9.3	2E-62	AT1G73620.1	Pathogenesis-related thaumatin
Eucgr.K01196	−7.6	2E-62	AT1G78570.1	rhamnose biosynthesis 1
Eucgr.K01402	37.9	3E-62	AT5G66780.1	unknown protein
CUFF.15800	37.9	6E-62	AT4G16260.1	Glycosyl hydrolase
Eucgr.H02579	−9.6	1E-61	AT5G09360.1	laccase 14
Eucgr.A01095	−7.5	2E-61	AT2G05790.1	O-Glycosyl hydrolases
Eucgr.K00883	−7.1	7E-61	AT4G03210.2	xyloglucan endotransglucosylase/hydrolase
Eucgr.K02657	−7.9	1E-60	AT1G75290.1	NAD(P)-binding Rossmann-fold superfamily protein
Eucgr.L01608	−8.3	8E-60	AT1G73270.1	serine carboxypeptidase-like 6
CUFF.31133	7.6	2E-59	AT1G43730.1	RNA-directed DNA polymerase (reverse transcriptase)
Eucgr.K01402	37.7	4E-59	AT5G66780.1	unknown protein
Eucgr.K03589	−6.8	4E-59	AT5G33370.1	GDSL-like Lipase/Acylhydrolase
Eucgr.H03385	8.4	6E-59	AT5G50850.1	Transketolase family protein
Eucgr.H00093	−7.8	4E-58	AT1G04680.1	Pectin lyase-like
Eucgr.A00203	−7.4	5E-58	AT3G16910.1	acyl-activating enzyme 7
Eucgr.F03878	7.9	6E-58	AT1G15330.1	Cystathionine beta-synthase
CUFF.38682	6.8	9E-58		no-hit
Eucgr.J01348	7.7	1E-57	AT5G15250.2	FTSH protease 6
Eucgr.B03992	−7.2	1E-57	AT5G49730.1	ferric reduction oxidase 6
Eucgr.E04218	−8.2	2E-56	AT5G09360.1	laccase 14
Eucgr.H00118	7.9	4E-56	AT1G04560.1	AWPM-19-like
Eucgr.I00519	−6.3	5E-56	AT4G33510.1	3-deoxy-d-arabino-heptulosonate 7-phosphate synthase
Eucgr.B02376	6.8	6E-56	AT3G46230.1	heat shock protein 17.4
Eucgr.F04203	9.4	6E-56	AT5G50080.1	ethylene response factor 110
Eucgr.G01115	−7.5	2E-55	AT4G12910.1	serine carboxypeptidase-like 20
Eucgr.A02790	6.3	3E-55	AT5G52300.2	CAP160 protein
Eucgr.C01388	−6.5	5E-55	AT2G10940.2	Bifunctional inhibitor/lipid-transfer protein
Eucgr.H04428	−6.3	2E-54	AT3G06350.1	dehydroquinate dehydratase, putative / shikimate dehydrogenase
Eucgr.I02267	7.0	4E-54	AT3G12500.1	basic chitinase
Eucgr.B03602	−7.2	5E-54	AT3G13510.1	Unknown Function
Eucgr.C00148	−9.3	5E-53	AT3G10450.2	serine carboxypeptidase-like 7

^1^The Arabidosis Information Resource.

Gene models predicted from reference-guided mapping were BLAST searched against Arabidopsis protein data base. Arabidopsis homologs of top significant genes are presented. *E. grandis* gene names are used when the predicted genes are mapped to *E. grandis* gene coordinates otherwise the predicted gene names are used with a pre-fix ‘CUFF’. “CUFF” gene coordinates, read counts and gene identities of all significantly (P < 0.05; bonferroni correction) differentially expressed transcripts are presented in the Additional file 2.

### Gene Ontology enrichment analysis

In order to determine the biological function of differentially expressed genes between control (C1) and stress (S1) treatments, gene ontology (GO) based enrichment tests were performed. The top most significantly differentially expressed genes (2642 genes; FDR <1.0e-10) were tested for enrichment using a web-based tool (GOMiner). *Arabidopsis* homologs of the predicted gene models were obtained by BLAST searches. A total of 175 gene categories were found to be significantly enriched among the genes that were differentially expressed between control (C1) and stress (S1) treatments. Of these, 140 categories were down-regulated, while 35 categories were up-regulated under stress treatment. Within the categories that were up-regulated, most of them were involved in stress response. For example, four of the most significantly enriched gene categories are response to chemical, temperature, heat and abiotic stress stimulus (Table 4). Similarly most of the down-regulated genes belonged to gene categories involved in metabolic processes and cell wall organisation (Table 4).

**Table 4:** Gene ontology based classification of differentially expressed genes

**GO CATEGORY**	**TOTAL GENES**	**CHANGED GENES**
**up-regulated**		
GO:0042221_response_to_chemical_stimulus	1271	123
GO:0050896_response_to_stimulus	2445	191
GO:0009266_response_to_temperature_stimulus	288	46
GO:0009408_response_to_heat	114	28
GO:0009628_response_to_abiotic_stimulus	974	94
GO:0006950_response_to_stress	1398	119
GO:0009644_response_to_high_light_intensity	42	16
GO:0009642_response_to_light_intensity	63	18
GO:0000302_response_to_reactive_oxygen_species	55	16
GO:0006979_response_to_oxidative_stress	178	29
GO:0042542_response_to_hydrogen_peroxide	36	13
GO:0009725_response_to_hormone_stimulus	551	55
GO:0010033_response_to_organic_substance	752	68
GO:0009415_response_to_water	149	24
GO:0009719_response_to_endogenous_stimulus	593	56
GO:0009737_response_to_abscisic_acid_stimulus	254	32
GO:0009414_response_to_water_deprivation	144	22
GO:0010035_response_to_inorganic_substance	360	38
GO:0006970_response_to_osmotic_stress	289	31
GO:0009651_response_to_salt_stress	269	28
**Down-regulated**		
GO:0009698_phenylpropanoid_metabolic_process	111	39
GO:0009699_phenylpropanoid_biosynthetic_process	83	32
GO:0019748_secondary_metabolic_process	230	57
GO:0007167_enzyme_linked_receptor_protein_signaling	94	31
GO:0007169_transmembrane_receptor_protein_tyrosine_kinase_signaling_pathway	94	31
GO:0007166_cell_surface_receptor_linked_signaling	113	32
GO:0009813_flavonoid_biosynthetic_process	44	19
GO:0019438_aromatic_compound_biosynthetic_process	151	37
GO:0006725_cellular_aromatic_compound_metabolic_proces	227	48
GO:0009812_flavonoid_metabolic_process	53	20
GO:0006629_lipid_metabolic_process	484	80
GO:0071555_cell_wall_organization	81	24
GO:0009653_anatomical_structure_morphogenesis	398	66
GO:0071554_cell_wall_organization_or_biogenesis	138	32
GO:0009664_plant-type_cell_wall_organization	38	15
GO:0042545_cell_wall_modification	60	19
GO:0008610_lipid_biosynthetic_process	259	47
GO:0007047_cellular_cell_wall_organization	41	15
GO:0071669_plant-type_cell_wall_organization_or_biogenesis	75	21
GO:0009827_plant-type_cell_wall_modification	19	10
GO:0070882_cellular_cell_wall_organization_or_biogenesis	89	23
GO:0048869_cellular_developmental_process	323	54
GO:0009808_lignin_metabolic_process	35	13

Gene ontology categories of up-regulated and down-regulated genes under water stress conditions (S1) among the most significantly differentiated transcripts. Total genes is the number of genes in a category, changed genes are the number of gene showed differential expression and FDR (<0.0001) is the one-sided Fisher’s exact *p* value corrected for multiple comparisons.

### Identification of growth related genes

To identify genes relating to growth and development we compared the gene expression between five plants from each population sampled at the beginning of the treatment (C0) and the same five plants sampled at the end of the treatment(C1). Gene expression analysis revealed a total of 3582 genes with significant (FDR < 0.01) differential expression between C0 and C1 samples. To study the expression patterns of these genes under stress conditions we compared the expression of significant genes (FDR < 0.01) from this analysis with those showing significant differential expression between control (C1) and stress (S1) treatments. In total there were 2225 genes common between the two analyses. A significant and negative correlation (*P* < 0.001; *r* = −0.37) was observed in log fold change of the gene expression between the two analyses indicating down regulation of several of the growth genes under stress treatment. Genes showing large fold changes in C1 versus S1 and C0 versus C1 comparison are shown in Table 5. While several photosynthetic and metabolic process related genes exhibited opposite signs in fold change, *basic chitinase*, *NAC* transcription factor and *homeo box* genes exhibited positive sign between the two comparisons. Gene ontology analysis reflected the down regulation of growth genes under stress conditions. Several metabolic process related gene categories such as ‘phenylpropanoid_metabolic_process’, ‘secondary_metabolic_process’ and ‘flavonoid_biosynthetic_process’ were up-regulated in C0 versus C1 comparison (Additional file 4) and the same gene categories were down-regulated in C1 versus S1 comparison (Table 4). However several stress response gene categories were up-regulated under both the comparisons.

**Table 5:** Comparison of fold changes in gene expression between different treatments

**Gene_id**	**LogFC**^**1**^	**LogFC**	**AT_accession**	**TAIR gene**
	**C0 vs C1**	**C1 vs S1**		**annotation**
CUFF.28412	−31.9	37.5	AT4G27450.1	Aluminium_induced
Eucgr.J00493	−34.4	36.4	AT2G27510.1	ferredoxin3
Eucgr.G01843	−31.8	34.3	AT5G45180.1	Flavin-binding
CUFF.8283	−31.2	31.8		unknown
Eucgr.F00644	−3.9	8.6		unknown
Eucgr.H04038	−3.0	7.4	AT3G62420.1	basicregion/leucinezipper
Eucgr.D00606	−2.4	7.2	AT1G28330.4	dormancy-associated
Eucgr.I02271	2.4	7.1	AT3G12500.1	basicchitinase
Eucgr.F02733	−5.4	7.1	AT1G78780.2	pathogenesis-related
Eucgr.H00163	−2.8	6.9	AT2G38940.1	phosphatetransporter
Eucgr.C03715	−4.2	6.7		unknown
Eucgr.E03257	−4.4	6.5	AT2G33830.2	Dormancy/auxinassociated
Eucgr.B03987	−2.9	6.4	AT1G60470.1	galactinolsynthase4
Eucgr.F01093	1.9	6.2	AT4G27410.3	NAC_transcriptional_regulator
Eucgr.B01275	−4.9	6.2	AT5G18600.1	Thioredoxin
Eucgr.D02645	3.2	6.0	AT3G61890.1	homeobox12
Eucgr.L01022	−4.2	5.8	ATCG00020.1	photosystem-II_reaction_center_protein
Eucgr.C03715	−3.1	5.8	AT5G56550.1	oxidative_stress3
Eucgr.J00639	−1.9	5.8	AT5G21170.2	5'-AMP-activated_protein_kinase
Eucgr.A02070	−1.8	5.8	AT3G04070.1	NAC_domain_containing_protein
Eucgr.F00195	3.4	−33.9	AT5G42830.1	acyl-transferase_family_protein
Eucgr.C04029	2.7	−33.9	AT2G24800.1	Peroxidase
Eucgr.A02214	3.5	−34.1	AT1G28110.2	serine_carboxypeptidase-like
Eucgr.C01020	5.0	−34.1		unknown
Eucgr.D02390	5.6	−34.1	AT5G49330.1	myb_domain_protein
Eucgr.F02915	4.4	−34.3		Novel
Eucgr.F03575	1.4	−34.3	AT1G32860.1	Glycosyl hydrolase
Eucgr.A02965	3.0	−34.4	AT5G17050.1	UDP-glucosyl_transferase
Eucgr.A02209	4.6	−34.4	AT1G28110.2	serine_carboxy_peptidase-like45
Eucgr.B03602	1.3	−34.4		unknown
Eucgr.A02209	3.8	−34.8	AT2G33530.1	serine_carboxypeptidase-like
Eucgr.C03801	4.8	−34.9	AT1G67290.1	glyoxaloxidase-related
Eucgr.A01768	2.2	−35.3	AT4G03270.1	Cyclin
Eucgr.I01041	5.5	−35.3	AT3G10450.2	serine_carboxypeptidase-like7
Eucgr.K02440	1.4	−35.5	AT3G53190.1	Pectinlyase-like
Eucgr.D02610	3.7	−35.6	AT1G73880.1	UDP-glucosyl_transferase
CUFF.12241	3.4	−35.7		unknown
Eucgr.L02741	5.4	−36.1	AT5G17040.1	UDP-Glycosyltransferase
Eucgr.A01963	5.8	−36.5	AT2G42840.1	protodermal_factor
Eucgr.F02646	3.4	−38.1	AT5G09360.1	laccase14

^1^logFC- log fold change.

Fold changes in the significantly differentiated genes between samples collected at the beginning (C0) and at the end of the experiment (C1) are compared with the fold changes in the significantly differentiated genes between control (C1) and stress (S1) treatments. *E. grandis* gene names are used when the predicted genes are mapped to *E. grandis* gene coordinates. Otherwise the predicted gene names are used with a pre-fix ‘CUFF’.

### Differential allelic expression

To study the regulatory variants responding to water stress treatment we measured allelic expression. For this the ten individuals sampled at the beginning of the treatment (S0) and the same ten individual sampled at the end of the stress treatment (S1) were used. Allelic expression of an individual should remain the same even when the total expression of a gene changes. Any change in the allelic expression may indicate the influence of regulatory variants. We observed several SNPs as ten individuals in each population were sequenced. To increase the coverage and confidence of the SNP calls, we combined the reads of the three populations from each treatment (30 trees from each treatment). Using a minimum coverage of 8 reads and a minimum frequency of 0.01, we identified 298,561 SNPs within S0 samples and 483,116 SNPs within the same samples under the stress treatment (S1). There were 196,375 SNPs common to both treatments. Most of the unique SNPs from either treatment generally had low coverage. Allele frequency differences between S0 and S1 treatments were used to identify differential allelic expression (DAE). This analysis revealed 2737 (FDR, P <0.05) SNPs (from 1261 genes) with significant differences in allelic expression between the two treatments (Additional file 5). Among these SNPs 68% were transition substitutions while 32% were transversion substitutions. *Chitinase*, *zinc finger*, *plastocynin* and *cellulose synthase* had large differences in allelic expression between the two treatments (Table 6). Allelic expression of 52% of SNPs (1427 SNPs) correlated with differential gene expression suggesting that these may be the *cis*-acting regulatory variants controlling gene expression. Genes with significant differences in allelic expression and total gene expression include *Chitinase*, *heat repeat containing protein*, and *Dehydrin*. Allelic expression of the remaining 48% of the SNPs (1310 SNPs) did not correlate with total gene expression. Several heat shock protein genes were present among this group (Additional file 5). The number of variants showing differential allelic expression was generally biased towards genes with high coverage and alleles with large expression differences between the treatments.

**Table 6:** Differential allelic expression

**Gene_id**	**SNP**	**Control**			**Stress**			**SNP**	**TAIR gene**
	**position**	**Allele-A**	**Allele-B**	**Freq**^**1**^	**Allele-A**	**Allele-B**	**Freq**	**Annotation**	**Annotation**
Eucgr.I01495	25151384	36	1	0.97	15	589	0.02	syn	basic chitinase
Eucgr.I02395	34587565	10	3	0.77	40	85	0.32	5'UTR	heat repeat-containing
Eucgr.I02395	34586749	8	16	0.33	177	6804	0.03	non-syn	dehydrin lea
Eucgr.F00644	8551578	5	44	0.10	56	2036	0.03	non-syn	GA requiring 3
Eucgr.D00606	11184666	6	3	0.67	33	266	0.11	syn	dormancy-associated
Eucgr.B03987	63762855	22	25	0.47	111	430	0.21	3'UTR	glycosyl transferase
Eucgr.B03987	63762861	20	29	0.41	96	382	0.20	3'UTR	glycosyl transferase
Eucgr.A02790	38441055	8	2	0.80	121	579	0.17	non-syn	low-temperature-induced
Eucgr.A02790	38441184	4	11	0.27	31	738	0.04	non-syn	low-temperature-induced
Eucgr.C01388	22208472	1114	155	0.88	8	7	0.53	syn	lipid transfer protein
Eucgr.I01393	24399270	28	1	0.97	39	158	0.20	non-syn	dnaj heat shock
Eucgr.I01393	24399277	28	3	0.90	40	162	0.20	non-syn	dnaj heat shock
Eucgr.I01393	24399018	4	6	0.40	205	11	0.95	non-syn	dnaj heat shock
Eucgr.C01031	16478607	7	6	0.54	101	748	0.12	syn	unknown protein
Eucgr.H00189	1874619	7	1	0.88	6	94	0.06	syn	cellulose synthase
Eucgr.G01188	19918572	5	12	0.29	24	390	0.06	syn	ethylene-dependent
Eucgr.K01389	17036132	49	19	0.72	259	1030	0.20	syn	xerico
Eucgr.K01389	17035852	7	53	0.12	34	1275	0.03	5'UTR	xerico
Eucgr.K01389	17035854	21	34	0.38	797	547	0.59	5'UTR	xerico
Eucgr.K01389	17036141	16	51	0.24	817	386	0.68	syn	xerico
Eucgr.E03875	67744315	114	123	0.48	6	28	0.18	non-syn	methyltransferase
Eucgr.C02590	49489875	35	1	0.97	87	324	0.21	non-syn	cipk6
Eucgr.C02590	49489315	14	0	1.00	26	62	0.30	5'UTR	cipk6
Eucgr.C02590	49489916	54	1	0.98	153	271	0.36	non-syn	cipk6
Eucgr.C02590	49489924	55	2	0.96	154	242	0.39	syn	cipk6
Eucgr.C02590	49489939	15	7	0.68	39	211	0.16	syn	cipk6
Eucgr.A02077	31568814	24	22	0.52	177	435	0.29	non-syn	oxidoreductase
Eucgr.I01579	25696053	36	5	0.88	10	18	0.36	syn	naringenin-chalcone synthase
Eucgr.I01579	25696038	30	4	0.88	15	17	0.47	syn	naringenin-chalcone synthase

^1^Freq – Frequency of allele-A.

SNPs with significant differential allelic expression between samples at the beginning (S0) and at the end of stress (S1) treatment. Genes with these SNPs also showed significant differential gene expression between control (C1) and stress (S1) treatments. *E. grandis* genes overlapping SNP positions are shown. Gene annotations are based on *Arabidopsis* homologs of the *Eucalyptus* transcripts. More details are shown in Additional file 5.

Using *E. grandis* gene annotations we classified the SNPs as three-prime, synonymous, nonsynonymous, five-prime and intronic SNPs. Synonymous and nonsynonymous SNPs were annotated using PoPoolation package [25]. While most of the SNPs were from coding regions, there were however several SNPs from intron regions (Table 6 and Additional file 5) suggesting that some of these SNPs may be from unspliced pre-mRNA. The intronic SNPs may also represent incomplete annotations of *E. grandis*. Ten of the intronic SNPs were within the splice sites.

### GO analysis of genes showing differential allelic expression

We used GO enrichment analysis to identify the functional categories enriched among the genes that showed significant differential allelic expression. GO enrichment tests were performed separately for genes that showed significant differential allelic expression as well as total gene expression between control (C1) and stress (S1) treatments and genes that showed only significant differential allelic expression but similar total gene expression between control and stress treatments. Genes that showed both allelic and total gene expression were enriched in stress and metabolic process gene categories (Additional file 6) as identified previously (Table 4). Interestingly, several stress-related gene categories (*e.g*. response to abiotic, salt and osmotic stress) were also enriched among the genes that showed differential allelic expression but no change in total gene expression (Additional file 6).

### Identification of genes under selection

To study the evolutionary selection patterns among the genes we analysed the nonsynonymous to synonymous substitution (Ka/Ks) ratios. To estimate the Ka/Ks ratios we combined the reads from all the populations before and after the stress treatment. We identified 194855 SNPs from coding regions of 13,719 genes using ‘PoPoolation’package[25]. These SNPs were annotated as nonsynonymous or synonymous using the ‘PoPoolation’ package. Annotations of these variants were further confirmed by visually inspecting the tracks in integrative genomics viewer IGV [26]. The proportion of nonsynonymous to synonymous mutation rates (Ka/Ks) among the genes has ranged from 0.05 to 5.9 with a mean of 0.39 among 13,719 genes (Additional file 7). Genes with Ka/Ks ratios below 0.5 were treated as under purifying selection while gene with Ka/Ks ratios above 1.5 were treated as under positive selection. Most of the genes (77 %, 10630 out of 13719genes) were under negative selection with the Ka/Ks ratios below 0.50. In contrast the number of genes under positive selection or under diversifying selection was small. Only 2% of the genes (287 out of 13719) were under positive selection with Ka/Ks ratios above 1.5. To identify the gene categories enriched among the genes we conducted GO enrichment tests separately for negatively and positively selected genes. While several gene categories relating to different biological processes were enriched among the negatively selected genes (Ka/Ks ratios < 0.20; Additional file 8), gene categories enriched among the positively selected genes were primarily related to apoptosis and cell death (Table 7).

**Table 7:** Enrichment of functional gene categories among positively selected genes

**GO CATEGORY**	**TOTAL GENES**	**CHANGED GENES**	**FDR**
GO:0006915_apoptosis	32	7	0.00
GO:0009404_toxin_metabolic_process	13	4	0.01
GO:0009407_toxin_catabolic_process	13	4	0.01
GO:0012501_programmed_cell_death	68	7	0.02
GO:0008219_cell_death	84	7	0.04
GO:0016265_death	84	7	0.04
GO:0006952_defense_response	254	12	0.08

### Differential gene and allelic expression of positively selected genes

Ninety genes which showed differential expressed between control (C1) and stress (S1) treatments were among the positively selected genes with Ka/Ks ratios more than 1.5 (Table 8). While several known genes and drought stress related transcription factors such as *NAC*, *ERF1* and *WRKY* were among the positively selected and differentially expressed genes there were however several unknown genes among the positively selected genes showing differential expression. Twenty seven SNPs from 17 positively selected genes showed differential allelic expression between S0 and S1 samples (Additional file 9). Of the 27 SNPs with differential allelic expression, 78% (21 out of 27) of them were nonsynonymous. Of the 17 genes which showed differential allelic -expression, four genes were differentially expressed between control (C1) and stress (S1) treatments. In three SNPs from two genes (LEA, and ketoreductase) expression of one of the two alleles was completely suppressed in S0 samples while both the alleles were expressed in S1 samples.

**Table 8:** Positively selected genes with differential gene expression between control (C1) and stress (S1) treatments

**Gene_id**	**Non-syn_length**^**1**^	**Syn_length**^**2**^	**Non-syn**^**3**^	**Syn**^**4**^	**Ka/Ks**	**FDR**	**TAIR annotation**
Eucgr.B02730	258	120	11	0	5.6	7E-16	no-hit
Eucgr.E04298	494	194	10	0	4.3	0.00	unknown
Eucgr.J02063	240	111	15	1	3.7	8E-09	Histone
Eucgr.H04702	312	126	8	0	3.6	8E-12	beta-ketoacyl reductase 1
Eucgr.B02265	353	166	6	0	3.3	2E-05	pleckstrin homologue 1
Eucgr.F04064	129	60	6	0	3.3	0.01	histone acetyltransferase
Eucgr.H04620	312	141	6	0	3.2	6E-13	unknown
Eucgr.D01191	841	398	12	1	3.1	0.01	Ubiquitin-conjugating enzyme
Eucgr.D01723	537	231	28	3	3.1	3E-11	glycine-rich protein
Eucgr.C02248	151	65	6	0	3	0.00	unknown
Eucgr.A01862	419	187	12	1	2.9	0.00	SAM
Eucgr.H03577	348	153	12	1	2.9	0.01	nuclear factor Y
Eucgr.D01717	385	152	6	0	2.8	0.00	Development and Cell Death
Eucgr.B02882	357	159	5	0	2.7	0.00	unknown
Eucgr.G00701	103	47	5	0	2.7	0.01	no-hit
Eucgr.K01312	362	163	5	0	2.7	7E-07	Late embryogenesis abundant
Eucgr.J03180	349	149	5	0	2.6	0.02	unknown
Eucgr.B03466	405	171	11	1	2.5	0.04	unknown
Eucgr.F00424	426	177	11	1	2.5	0.01	nucleoside triphosphate hydrolase
Eucgr.I01743	145	59	5	0	2.5	0.00	unknown
Eucgr.G01464	61	29	4	0	2.4	0.00	no-hit
Eucgr.C00195	311	142	4	0	2.3	0.03	glutamine dumper
Eucgr.K02172	796	329	27	4	2.3	2E-05	proteolysis
Eucgr.D01225	367	161	4	0	2.2	0.00	Cupredoxin superfamily protein
Eucgr.K01587	505	233	18	3	2.2	0.00	Calcium-binding EF-hand family protein
Eucgr.G02486	272	112	4	0	2.1	1E-21	NAC-like
Eucgr.H04886	638	265	9	1	2.1	0.03	A20/AN1-like zinc finger
Eucgr.I01744	123	51	4	0	2.1	1E-06	no-hit
Eucgr.L02163	143	61	9	1	2.1	0.00	TRAF-like
Eucgr.C01903	219	90	4	0	2	7E-07	UDP-glucosyl transferase
Eucgr.F01425	160	65	4	0	2	2E-10	glutathione S-transferase
Eucgr.I01164	351	156	8	1	2	1E-05	CXE12
Eucgr.C02013	720	331	28	6	1.9	1E-10	unknown
Eucgr.E01742	103	48	3	0	1.9	0.01	agenet domain-containing
Eucgr.G00935	80	31	4	0	1.9	0.01	no-hit
Eucgr.I02449	307	134	12	2	1.9	0.02	no-hit
Eucgr.J03046	168	78	3	0	1.9	2E-22	pleiotropic drug resistance
Eucgr.A02694	128	59	3	0	1.8	5E-17	UDP-glucosyl transferase
Eucgr.B01852	339	138	12	2	1.8	1E-34	soybean gene regulated by cold-2
Eucgr.B02741	317	142	7	1	1.8	2E-42	heat shock protein 17.4
Eucgr.D02113	186	84	3	0	1.8	1E-11	ethylene responsive element binding factor
Eucgr.E00479	58	26	3	0	1.8	0.00	unknown
Eucgr.F03056	1150	473	12	2	1.8	0.00	NTH/VHS family
Eucgr.H02910	254	106	12	2	1.8	0.01	Calcium-binding EF-hand family
Eucgr.A02080	67	29	3	0	1.7	0.00	unknown
Eucgr.B03040	176	76	3	0	1.7	9E-06	unknown
Eucgr.D00574	477	208	3	0	1.7	2E-26	GDSL-like Lipase/Acylhydrolase
Eucgr.D01956	605	250	7	1	1.7	2E-18	cytochrome P450
Eucgr.F03511	188	79	3	0	1.7	0.05	no-hit
Eucgr.H03533	55	23	3	0	1.7	0.01	Haloacid dehalogenase-like hydrolase
Eucgr.I00325	221	91	3	0	1.7	0.03	disproportionating enzyme
Eucgr.L03100	466	194	3	0	1.7	3E-08	cytochrome P450
Eucgr.B03756	184	74	3	0	1.6	1E-12	beta-6 tubulin
Eucgr.C00342	193	80	14	3	1.6	0.00	cytochrome P450
Eucgr.D00603	111	46	3	0	1.6	0.00	Yippee family
Eucgr.E00452	400	164	3	0	1.6	0.00	unknown
Eucgr.E03921	254	107	14	3	1.6	4E-25	Cystatin/monellin
Eucgr.E04006	104	43	3	0	1.6	0.01	pyridoxal phosphate-dependent
Eucgr.F00810	31	17	2	0	1.6	0.01	RNI-like superfamily protein
Eucgr.F02734	205	83	3	0	1.6	0.03	unknown
Eucgr.G02336	616	269	14	3	1.6	0.00	unknown
Eucgr.G02823	394	159	3	0	1.6	0.02	para-aminobenzoate (PABA) synthase
Eucgr.G03355	724	311	18	4	1.6	1E-19	NAD(P)-binding Rossmann-fold
Eucgr.H00155	459	183	15	3	1.6	2E-13	glutathione S-transferase
Eucgr.H00996	111	45	3	0	1.6	3E-06	WRKY DNA-binding protein
Eucgr.H01946	252	99	3	0	1.6	2E-08	unknown
Eucgr.H04711	189	75	3	0	1.6	1E-12	beta-ketoacyl reductase
Eucgr.H05152	281	115	3	0	1.6	0.00	GA20OX1
Eucgr.K01016	236	106	13	3	1.6	6E-12	unknown
Eucgr.L03046	171	78	6	1	1.6	1E-07	no-hit
Eucgr.A01283	476	190	10	2	1.5	5E-06	far-red elongated hypocotyl
Eucgr.C02545	651	261	10	2	1.5	0.02	WRKY DNA-binding protein
Eucgr.C03738	53	19	3	0	1.5	0.00	no-hit
Eucgr.E00907	198	87	6	1	1.5	7E-07	NB-ARC domain-containing disease resistance
Eucgr.E02617	80	40	2	0	1.5	1E-07	SAM
Eucgr.E03833	453	195	6	1	1.5	6E-06	C2 calcium/lipid-binding
Eucgr.F00172	57	21	3	0	1.5	3E-05	cytochrome P450
Eucgr.F01135	1007	418	10	2	1.5	5E-09	NB-ARC domain-containing disease resistance
Eucgr.F01523	66	24	3	0	1.5	4E-07	no-hit
Eucgr.F01700	152	58	3	0	1.5	7E-13	sterol methyltransferase 2
Eucgr.F04454	254	97	3	0	1.5	0.00	unknown
Eucgr.G00423	159	69	6	1	1.5	4E-19	no-hit
Eucgr.G01862	673	317	15	4	1.5	3E-11	EXORDIUM like
Eucgr.H02734	30	15	2	0	1.5	5E-05	F-box family
Eucgr.I00883	829	362	36	10	1.5	8E-20	no-hit
Eucgr.I01025	2	1	2	0	1.5	0.02	photosystem II subunit O-2
Eucgr.J00894	163	62	3	0	1.5	1E-17	Heavy metal transport/detoxification superfamily protein
Eucgr.J02210	760	326	6	1	1.5	2E-07	Tetratricopeptide repeat
Eucgr.K01084	101	49	2	0	1.5	4E-05	protein kinase C substrate
Eucgr.L01514	81	30	3	0	1.5	0.00	unknown

^1^nonsynonymous length ^2^synonymous length ^3^nonsynonymous sites ^4^synonymous sites.

## Discussion

We observed several (> 2500) genes differentially expressed between control and water stress conditions. The large numbers of genes observed in this study compared to other studies could be due to the higher sensitivity of RNA-seq compared to microarray analysis. The high correlation in gene expression between three populations in both control and stress treatments may be due to the same factors that led to the similarity of physiological and biomass traits observed between the populations in both the treatments (Table 1).

### GO analysis reveals biologically relevant genes

Gene ontology-based tests revealed more than 100 gene categories enriched among the top most significantly differentially expressed genes. While several drought stress genes were induced by stress treatment, several cell wall and photosynthetic genes were down regulated under stress conditions. Several growth and development genes identified by comparing the control samples taken at two intervals were down regulated under stress treatment. Up-regulation of several metabolic process genes between the control samples and down regulation of these gens under stress treatment may reflect the reduction in growth under stress conditions suggesting that these genes play a role in normal plant growth and development. These genes may therefore be used as candidate genes for growth and biomass production.

In addition to the previously reported water stress related genes, we observed several novel and/or unknown genes showing differential expression between control and stress treatments. These form a new source of candidate genes for water stress tolerance. Functional analysis of these genes may reveal novel pathways of genes responding to water stress. The new gene models predicted with reference-guided mapping which are not present in *E. grandis* annotations (Additional file 2) may be useful for improving the annotations of *E. grandis* gene models.

### Differential allelic expression is common

In addition to studying gene expression patterns, RNA sequencing can also reveal differences in allelic expression. Allelic expression analysis can reveal functional regulatory variants. Within an individual both alleles are subjected to same environmental conditions and feedback control. Any bias in the expression of two alleles indicates presence of nearby *cis*-variants. In RNA sequencing experiments read counts at the polymorphic sites provide allelic abundance and simple statistical tests of differences in read counts at polymorphic sites allow the detection of biases in allelic expression [27]. Allelic expression imbalance (AEI) is generally measured by genotyping or sequencing SNPs in individual cDNA samples [28-30]. However, high throughput sequencing methods have recently been used for studying AEI [31,32]. While sequencing individual samples for AEI analysis is a powerful approach for detecting subtle differences in allelic expression, it is expensive to sequence individual samples separately. Next generation sequencing of pooled samples provides cost effective method for estimating allele frequencies at genome-wide scale [33]. Pooling and sequencing RNA samples is an efficient way to detect *cis*-regulatory polymorphisms at genome-wide scale [34]. Sequencing RNA samples pooled from 100 adipose and islet tissues of F_2_ mice, Babak *et al.*. [35] found several genes showing AEI. They found a significant overlap between the genes showing AEI and *cis*-eQTL genes obtained from microarray analysis of the same F_2_ population, indicating the robustness of this approach. While differential allelic expression from RNA sequencing of pooled samples may not indicate the presence of *cis*-acting variants, the correlation of allelic expression with total gene expression may indicate the presence of nearby *cis*-acting variants.

We used pooled RNA samples to identify SNPs in this study. Allelic expression of about 52 % of significant variants correlated with differential gene expression between control (C1) and stress (S1) treatments. These variants therefore may represent *cis*-acting regulatory variants controlling gene expression or these variants may occur in high LD with regulatory variants in adjacent intronic, untranslated and promoter sequences. Recent genome-wide association studies have demonstrated that genetic variation in regulatory regions is more important than coding regions in affecting complex traits [36]. Identification of regulatory polymorphisms is therefore crucial for understanding the control of complex traits.

Allelic expression was shown to influence gene expression and phenotype in several plant species. Drought stress was shown to induce allele specific expression in barley hybrids [37]. Allelic expression may also by caused by differential methylation of alleles. In a recent study we showed that a single nucleotide polymorphism (SNP) in a *cis*-regulatory element affects tree phenotypic traits (cellulose and pulp yield) through changes in allelic expression caused by differential methylation of alleles [38]. SNP markers within regulatory elements can therefore affect traits by influencing the expression of genes, and could potentially be used in breeding programs to improve complex traits such as drought tolerance, growth and wood quality traits.

Enrichment of several stress responsive gene categories among the genes showing DAE and similar total gene expression between control and stress treatments indicates that these variants may be the trans-acting variants or variants influenced by mutations to transcriptional network. Similar results were reported by Tuch *et al.*[32]. By comparing gene expression patterns between tumour and normal tissues they identified several genes with differential allelic expression but similar total gene expression between the two types of tissues. Gene ontology tests with allelically imbalanced genes indicated enrichment of several gene categories common to the set of differentially expressed genes between tumour and normal tissues. These results indicate that allelic expression analysis may be helpful in identifying candidate genes even when total gene expression differences between the treatments are subtle.

While sequencing pooled samples is a cost effective method, pooling different samples may however introduce different biases. To verify the allelic expression results from this study these SNPs need to be sequenced or genotyped in independent samples. Similarly, the pooling method used in this study does not allow for the detection of causal variants. Sequencing or genotyping of individual samples is required to identify the causal regulatory variants [34].

### Evolutionary signatures of selection among the genes

To explore the evolutionary selection patterns among the genes and to identify the mechanisms of natural selection under water stressed conditions we studied the selection signatures using Ka/Ks estimates. Most of the genes examined in this study are under negative or purifying selection with a mean Ka/Ks ratio of 0.39 (Additional file 7). Similar results were reported in *E. grandis*[19]. The average Ka/Ks ratio observed in that study was 0.30. In the previous study, Novaes *et al.*[19] have analysed 2001 genes while in the present more than 13,000 genes were analysed. This study thus provides genome-wide selection patterns among the genes expressed in the leaf tissue. Most of the protein coding genes in plants and animals are in general under purifying selection indicating that these genes may have central functions and nonsynonymous mutations affecting their function have been removed by purifying selection [39]. Gene ontology (GO) enrichment tests have revealed gene categories belonging to several biological processes were enriched among the negatively selected genes (Additional file 8). Similar results were reported in *E. grandis* where genes encoding protein translation were the most significantly enriched among negatively selected genes [19]. In the present study however apoptosis and cell death categories were significantly enriched among the positively selected genes (Table 7). Nielsen *et al.*. [40] have also reported that apoptosis related genes were under strong positive selection among 13,731 homologous genes between human and chimpanzee lineages.

Apoptosis involves removal of cells damaged by stresses or pathogen infections through programmed cell death. Several studies in plants have shown that disease resistance genes are under strong positive selection [41]. The role of apoptosis in defence mechanisms may be the reason for positive selection acting on genes relating apoptosis and cell death. Genes relating to stress particularly disease stress evolve rapidly to adapt to changing conditions. Maintaining different alleles will help the organisms to cope with the changing conditions [42].

The estimates of Ka/Ks ratios in the present study are influenced by coverage of the genes. The results presented here should therefore need to be further validated. Further studies using entire genome sequences of several closely related *Eucalyptus* species should improve the knowledge of selection patterns among different genes. With the availability of *Eucalyptus* reference genome sequence and the development of improved sequence analysis tools such genome-wide comparisons are now possible.

## Conclusions

We identified numerous genes that are differentially expressed between control and water stressed *E. camaldulensis* seedlings. In addition to the previously characterised genes we observed several novel and/or unknown genes showing differential expression. Functional analysis of these genes may provide novel insights into the genetic control of drought tolerance. We also identified several SNPs in the differentially expressed genes with allelic expression of several of these variants correlating with total gene expression. The correlation of allelic expression with total gene expression indicates that these variants may be the *cis*-acting regulatory variants or in LD with such variants. Analysis of the selection patterns revealed enrichment of apoptosis and cell death categories among the positively selected genes. The variants identified from differential allelic expression form a valuable resource for further studies such as association studies to identify markers for drought resistance. Through this study we show that RNA-seq can be used to reveal functional markers and evolutionary selection patterns in addition to candidate genes.

## Methods

### Glass house experiments

*Eucalyptus camaldulensis* seeds were sourced from three provenances with contrasting climates: Petford (humid tropics), Katherine (dry tropics) and Mt.Isa (semi-arid tropics). Fifteen genotypes from each provenance were grown in pots (approximately 8 l) in a glass house in April 2009. Temperatures in the glasshouse were maintained at 15°C minimum and 30°C maximum. The soil surface in the pots was covered with plastic beads to reduce evaporation. After growing the plants for five months water stress was imposed on ten genotypes by maintaining the pots at 30% of field capacity for two months. The other five genotypes were watered to field capacity (controls). Stomatal conductance, photosynthetic assimilation rates and pre-dawn water potential were measured just before imposition of water stress (8^th^ of August) and during the stress period at periodic intervals from both stressed and control plants. At the same time leaf samples were taken for RNA extraction from both stressed and control plants and immediately stored at −80°C. Water usage was monitored throughout the experiment by weighing the pots. Two pots containing soil but no plants were also weighed, to estimate water loss by evaporation. All plants were harvested two months after imposing the stress treatment (10^th^ of October). Harvested plants were separated into roots and shoots, oven dried at 70°C and biomass measurements were taken.

### Physiological trait measurements

Physiological measurements were taken during the experiment, at three time points: (1) immediately prior to the imposition of water stress (8/8/2009), (2) 30 days after the imposition of water stress (11/9/2009), and (3) 52 days after the imposition of water stress (9/10/2009). Pre-dawn and mid-day water potentials and osmotic potentials were measured on fully expanded young leaves using psychrometers. Measurements of stomatal conductance were taken ten days after the imposition of stress treatment using a hand-held porometer. To determine the maximum conductance, diurnal changes in stomatal conductance were measured on three plants over three days. From this analysis it was determined that maximum conductance occurred between 11.00 AM and 1.00 PM. Leaf area of all plants was measured at final harvest.

Two-way analysis of variance (ANOVA) was used to test the effects of population, treatment and the interaction between treatment and population on all the traits measured using ANOVA functions in ‘R’ statistical package. Pair-wise differences between the populations for the traits were tested with Tukey’s post-hoc tests.

### RNA isolation

Each population of 15 seedlings was divided into two groups of ten and five seedlings before collecting RNA samples. Two leaf samples from each seedling were taken before noon just before the imposition of stress on 8^th^ of August. Leaf samples from ten and five seedlings of each population were bulked separately before isolating RNA. Leaf samples from 10 seedlings collected at the start of the treatment were designated as “S0” and the leaf samples from five plants taken at beginning of the treatment were designated as “C0”. Similarly, two leaves from each plant were collected before noon at the end of the stress treatment on 9^th^ of October. Leaf samples taken from the ten seedlings under stress treatment were designated as “S1” and the leaf samples taken from the five control plants at the end of the treatment were designated as “C1”. Equal amounts of leaf tissue from each population were bulked before extracting RNA. In total RNA was isolated from 12 bulks, six bulks before stress treatment (S0, C0) and six bulks at the end of stress treatment (S1, C1). RNA was isolated using Chang *et al.*, [43] method and concentrations were measured using Qubit florometer. Purified RNA samples were sent to GeneWorks for high throughput illumina sequencing. RNA sequencing libraries were prepared using total RNA. In total, five lanes of a flow cell were used for sequencing 12 libraries. Samples were sequenced with 65 base single end reads.

#### Read mapping

Reference-guided transcriptome mapping was performed with the reads from high throughput sequencing. Reads were assembled using the reference genome sequence of *E. grandis* but without using the *E. grandis* gene annotations i.e., annotations were developed *ab initio* for *E. camaldulensis*. *E. grandis* gene models mapping to *E. camaldulensis* predicted gene models were obtained using a BED file of the predicted gene coordinates in BEDTools package [44].

The draft genome of *Eucalyptus grandis* (http://web.up.ac.za/eucagen) was used for reference-guided mapping of transcriptome sequencing reads. Sequencing reads from all 12 transcriptome libraries were first pooled and mapped to the *Eucalyptus* genome sequence scaffolds using the Bowtie [45] and TopHat [46] software packages. Bowtie was used to index the reference genome and to map sequencing reads to the indexed genome, and TopHat identified potential exon splice junctions, and mapped sequencing reads to these junctions. TopHat was run with the default parameters except for a maximum intron length of 5000 bp. The resulting alignment (in BAM file format) was used to generate transcript annotations (in gene transfer file, or GTF, format) with the Cufflinks [47] software package. Cufflinks was run with the default parameters without supplying any annotation file. Bias detection and correction to improve the accuracy of transcript abundance was used by supplying a multi fasta file of *E. grandis* genome. Secondly, sequencing reads from the individual libraries were mapped against the reference genome sequence with TopHat to obtain alignment files (BAM) for each of the 12 libraries. The BAM file from each library was analysed with the BEDTools [44] software package, which provided counts of reads mapping to different gene products (transcripts) that were represented in the annotation file (GTF). These read counts were used in statistical tests of differential expression between control and stress treatments. Read sequence and the read counts data are deposited in NCBI’s Gene Expression Omnibus and are accessible through GEO series accession number GSE39369.

### Analysis of differential gene expression

Differences in gene expression between different samples were tested with edgeR [48] and DESeq [49] packages using read counts from reference-guided mapping. Read counts from three populations were used as biological replicates in differential gene expression analysis. Genes expressed at very low levels (read counts < 10 across all six libraries) were not used in analysis of differential gene expression. The model used in edgeR for testing differential gene expression was based on a negative binomial distribution. Significance tests for differential expression were based on a modified exact test. A false discovery rate (FDR) of 0.01 was used for identifying differentially expressed genes. Similar to edgeR, DESeq also uses read counts for testing differential gene expression analysis. Variance stabilized data obtained with DESeq was used to generate the heatmaps of differentially expressed genes.

To study the biological significance of differentially expressed genes, gene ontology (GO) based enrichment tests were conducted using a web based tool GOMiner [50] (http://discover.nci.nih.gov/gominer/index.jsp). For this analysis *Arabidopsis* homologs of transcripts were obtained by BLAST searching the *Arabidopsis* protein database using blastx. BLAST search was run with the parameters of maximum high scoring segment pairs (HSP) of 100, expect value for matches of 10 and the default matrix of BLOSUM62. To identify *Arabidopsis* homologs for gene models predicted from reference-guided transcriptome mapping, gene sequences were extracted from the *Eucalyptus* reference genome sequence using gene coordinates from the gene annotation file (GTF) generated using the ‘Cufflinks’ package. The extracted gene sequences were BLAST searched with the *Arabidopsis* protein database. The identified *Arabidopsis* homologs were used in GO enrichment tests.

#### Identification of SNPs

To study allelic expression SNPs from ten seedlings before the treatment (S0) and the same ten seedlings after treatment (S1) were analysed. The BAM files generated from TopHat analysis were used for detecting SNPs. The BAM files were used in SAMTools [51] to produce pileup files containing SNP information. Pileup files generated from SAMtools were analysed with VarScan software [52] to count the reads mapping to each allele of a variant and to estimate the allele frequencies. The following options were used in VarScan to detect the SNPs. A minimum coverage of 8 reads mapping to variant sites, minimum base phred quality of 20 and a *P*-value of 0.05 were used for SNP calling. Reads from the three control treatment libraries (S0) and reads from the three stress treatment libraries (S1) were combined for detecting SNPs. Read counts of variant alleles from control (S0) and stress treatments (S1) were used in testing for differential allelic expression using chi-squared tests. Only consistent SNPs i.e. SNPs with the same alleles from both control and stress treatment were used in the differential allelic expression analysis. SNPs with a coverage of less than 20 reads in both the treatments were not used. Significance of the differential allelic expression was based on FDR (=0.05). The BEDTools package was used to identify gene features (from GTF file) as well as *E. grandis* genes overlapping SNPs.

### Identification of genes under selection

To study the selection patterns of genes we have estimated the proportion of nonsynonymous to synonymous substitutions (Ka/Ks). We used the PoPoolation[25] package to identify and to annotate SNP variants *i.e*. to determine if an SNP is nonsynonymous or synonymous This tool uses a pileup file generated from SAMTools and a gene annotation file of coding sequences (CDS) to identify and to annotate the SNP variants. We used a BAM file generated from combining the reads from before and after the stress treatments in SAMTools to generate the pileup file containing SNP information. We extracted the coding sequences (CDS) mapping to each gene by using *E. grandis* gene annotation file (gff3). We used a minimum coverage of 20 reads, a maximum coverage of 8000, minimum phred quality of 20 and a minimum allele count of 4 for identifying the variants. The maximum coverage was based on the observed maximum SNP coverage of 7961 reads with a minimum base quality of 20. The identity of the variants was further confirmed by visual inspection of the tracks in integrative genomics viewer (IGV)[26]. We uploaded the BAM files, the SNP position files and *E. grandis* gene annotation files (gff3) into IGV and visually inspected the variants from different genes to confirm the annotations. The identified nonsynonymous and synonymous SNPs were normalised by non-synonymous and synonymous lengths calculated using the ‘PoPoolation’ package. The average nonsynonymous length of each codon was calculated using transversion penalty of 6. The synonymous length was calculated as 3-nonsynonymous length. The Ka/Ks ratios were estimated following Novaes *et al.*[19] by adding a unit to both nonsynonymous and synonymous sites. To identify the gene categories enriched among the positively and negatively selected genes we conducted the GO tests by comparing the gene categories enriched among positively and negatively selected genes separately. To identify the gene categories enriched among the positively selected genes, all the genes with Ka/Ks ratios more than 1.5 were compared with the rest of the genes. Similarly to identify the genes enriched among the negatively selected genes, all the genes with Ka/Ks ratios less than 0.20 were compared with the rest of the genes. GOMiner package [50] was used for GO analysis of the selected genes.

## Abbreviations

SNP: Single nucleotide polymorphisms; RNA-seq: RNA sequencing; GO: Gene Ontology; DAE: Differential allelic expression; LD: Linkage disequilibrium.

## Competing interests

The authors declare that they have no competing interests.

## Authors’ contributions

BT, NS and SS conceived the research; BT designed and conducted the experiments; BT analysed the data, BT, NS and SS drafted the manuscript. All authors read and approved the final manuscript.

## Supplementary Material

Additional file 1 **Figure S1.** Comparison of biomass traits between treatments. Error bars are standard errors of mean (SEM). K-Katherine; M-Mt Isa; P-Petford. Click here for file

Additional file 2 Read counts and gene identities of the significantly (P ≤ 0.05; bonferroni correction) differentially expressed transcripts between control (C1) and stress (S1) treatments. Click here for file

Additional file 3 Pearson’s correlation coefficients among the reference-guided assembly read counts from 12 libraries. Click here for file

Additional file 4 Gene ontology analysis of differentially expressed transcripts between control samples collected at the beginning (C0) and at the end (C1) of the treatment. Click here for file

Additional file 5 Allele frequencies of the SNPs with significant differential allelic expression between samples collected at the beginning (S0) and at the end (S1) of stress treatments. Click here for file

Additional file 6 **Enrichment of functional gene categories among genes with differential allelic expression.** a). Gene categories enriched among genes showing differential allelic expression (between S0 and S1samples) and differential total gene expression between control (C1) and stress (S1) treatments. b). Gene categories enriched among genes showing differential allelic expression but similar total gene expression between control (C1) and stress (S1) treatments. FDR < 0.001. Click here for file

Additional file 7 **Distribution of Ka/Ks ratios among the genes.** Ka/Ks values are based on full length CDS gene annotations from *E. grandis*. Click here for file

Additional file 8 **Gene categories enriched among genes under purifying selection.** Genes with Ka/Ks ratio < 0.20 were used in GO analysis. Click here for file

Additional file 9 Differential allelic expression among positively selected genes. Click here for file
