# Supplementary figures and images for: Transcriptome sequencing of Eucalyptus camaldulensis seedlings subjected to water stress reveals functional single nucleotide polymorphisms and genes under selection
Source: BMC Genomics. 2012 Aug 1;13:364. doi: 10.1186/1471-2164-13-364 (PMC3472208; doi:10.1186/1471-2164-13-364)

Supplementary Figure S1

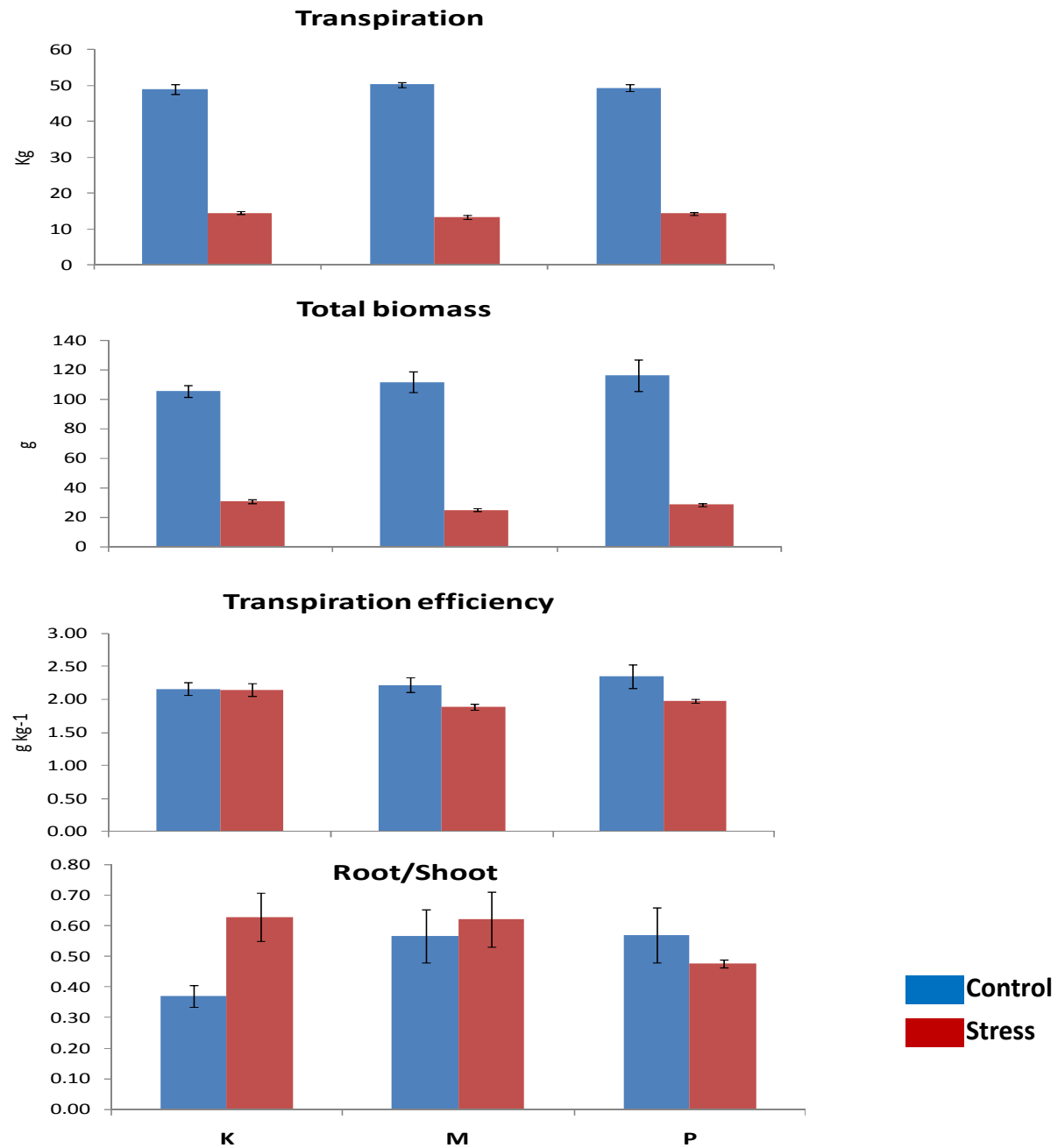

Supplement: Additional file 1 — Figure S1. Comparison of biomass traits between treatments. Error bars are standard errors of mean (SEM). K-Katherine; M-Mt Isa; P-Petford. [file 1471-2164-13-364-S1.pdf]

## Slide 1
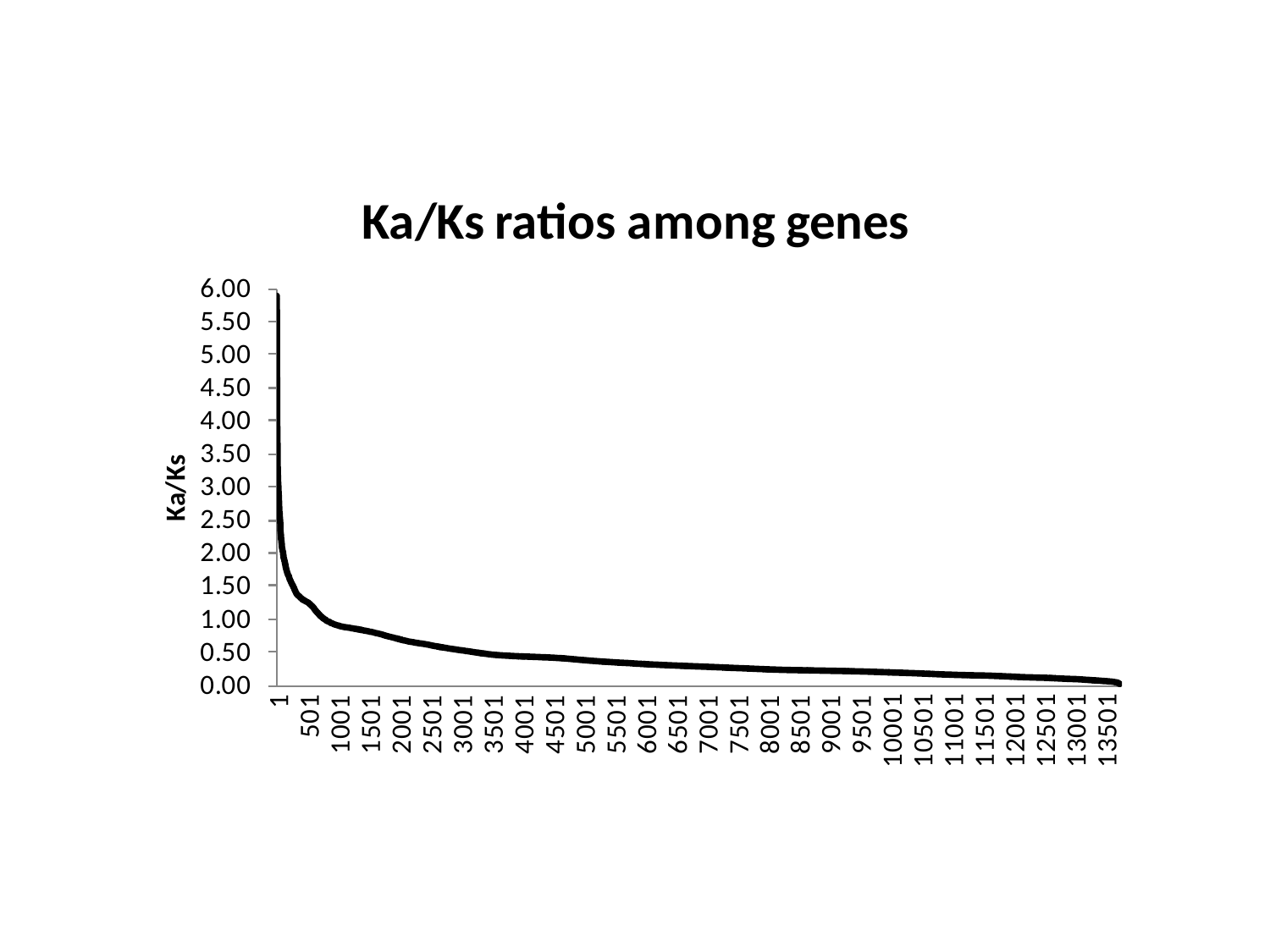

Supplement: Additional file 7 — Distribution of Ka/Ks ratios among the genes. Ka/Ks values are based on full length CDS gene annotations from E. grandis. [file 1471-2164-13-364-S7.ppt]
